# Continuous theta burst stimulation over left supplementary motor area facilitates auditory-vocal integration in individuals with Parkinson’s disease

**DOI:** 10.3389/fnagi.2022.948696

**Published:** 2022-08-16

**Authors:** Guangyan Dai, Meng Wang, Yongxue Li, Zhiqiang Guo, Jeffery A. Jones, Tingni Li, Yichen Chang, Emily Q. Wang, Ling Chen, Peng Liu, Xi Chen, Hanjun Liu

**Affiliations:** ^1^Department of Rehabilitation Medicine, The First Affiliated Hospital, Sun Yat-sen University, Guangzhou, China; ^2^Department of Radiology, The First Affiliated Hospital, Sun Yat-sen University, Guangzhou, China; ^3^School of Computer, Zhuhai College of Science and Technology, Zhuhai, China; ^4^Psychology Department and Laurier Centre for Cognitive Neuroscience, Wilfrid Laurier University, Waterloo, ON, Canada; ^5^Department of Communication Disorders and Sciences, RUSH University Medical Center, Chicago, IL, United States; ^6^Department of Neurology, The First Affiliated Hospital, Sun Yat-sen University, Guangzhou, China; ^7^Guangdong Provincial Key Laboratory of Brain Function and Disease, Zhongshan School of Medicine, Sun Yat-sen University, Guangzhou, China

**Keywords:** Parkinson’s disease, auditory feedback, vocal motor control, continuous theta burst stimulation, supplementary motor area

## Abstract

Accumulating evidence suggests that impairment in auditory-vocal integration characterized by abnormally enhanced vocal compensations for auditory feedback perturbations contributes to hypokinetic dysarthria in Parkinson’s disease (PD). However, treatment of this abnormality remains a challenge. The present study examined whether abnormalities in auditory-motor integration for vocal pitch regulation in PD can be modulated by neuronavigated continuous theta burst stimulation (c-TBS) over the left supplementary motor area (SMA). After receiving active or sham c-TBS over left SMA, 16 individuals with PD vocalized vowel sounds while hearing their own voice unexpectedly pitch-shifted two semitones upward or downward. A group of pairwise-matched healthy participants was recruited as controls. Their vocal responses and event-related potentials (ERPs) were measured and compared across the conditions. The results showed that applying c-TBS over left SMA led to smaller vocal responses paralleled by smaller P1 and P2 responses and larger N1 responses in individuals with PD. Major neural generators of reduced P2 responses were located in the right inferior and medial frontal gyrus, pre- and post-central gyrus, and insula. Moreover, suppressed vocal compensations were predicted by reduced P2 amplitudes and enhanced N1 amplitudes. Notably, abnormally enhanced vocal and P2 responses in individuals with PD were normalized by c-TBS over left SMA when compared to healthy controls. Our results provide the first causal evidence that abnormalities in auditory-motor control of vocal pitch production in PD can be modulated by c-TBS over left SMA, suggesting that it may be a promising non-invasive treatment for speech motor disorders in PD.

## Introduction

One prominent clinical feature of idiopathic Parkinson’s disease (PD) is hypokinetic dysarthria, which is characterized by a reduction in speech volume and pitch fluctuation, inconsistent rates of speech, and imprecise articulation (Duffy, 2005). These abnormalities in speech production occur in individuals with early-stage PD and deteriorate as the disease progresses (Rusz et al., 2013; Moreau and Pinto, 2019). Approximately, 70–90% of individuals with PD have speech motor disorders that limit their communication, social participation, and quality of life (Sapir et al., 2008). However, treatment of PD dysarthria remains a challenge. Despite the remarkable positive effects of pharmacological and surgical interventions on motor limb symptoms of PD, the effects of these treatments on PD dysarthria are commonly more deleterious than they are beneficial (Skodda et al., 2009). The Lee Silverman Voice Treatment (LSVT^®^ LOUD), a behavioral speech therapy designed for the treatment of PD hypophonia, has been shown to produce immediate and long-term (12–24 months) improvement in vocal loudness, pitch variability, and speech intelligibility (Ramig et al., 2001; Sapir et al., 2007). The application of this treatment, however, is limited by high-effort and intensive vocal training that cause many patients to discontinue treatment. Therefore, the development of other effective speech therapies for PD dysarthria remains an important goal.

Repetitive transcranial magnetic stimulation (rTMS) has emerged as an important tool in the experimental treatment of various psychiatric and neurological disorders by non-invasively modulating brain activity to change behaviors (Hallett, 2007). It is generally believed that high-frequency rTMS applied over the target site increases the excitability of cortical neurons, whereas low-frequency rTMS decreases it (Pascual-Leone et al., 1994; Chen and Seitz, 2001), although this relationship may vary as a function of stimulation intensity and individual variability (Fitzgerald et al., 2006; Lopez-Alonso et al., 2014). One meta-analysis showed that high-frequency rTMS over the primary motor cortex (M1) or low-frequency rTMS over the dorsolateral prefrontal cortex (DLPFC) produced moderate improvements in PD motor disorders (Chou et al., 2015). In contrast, studies investigating the efficacy of rTMS for PD dysarthria have found conflicting results. For example, a single session of high-frequency rTMS over the primary orofacial sensorimotor area (SM1) led to improved vocal pitch and loudness, tongue movement, and voice quality in individuals with PD (Dias et al., 2006; Eliasova et al., 2013), whereas high-frequency rTMS over the left DLPFC and left M1 hand area failed to do so (Dias et al., 2006; Hartelius et al., 2010; Eliasova et al., 2013). Brabenec et al. (2019) found that, following a single session of low-frequency rTMS over the right superior temporal gyrus (STG), individuals with PD exhibited increased variability of the second formant that was predicted by enhanced right STG activation during sentence reading. However, beneficial effects were not found when they received high-frequency rTMS over the right STG or SM1. Brabenec et al. (2021) subsequently found that following 10 sessions of low-frequency rTMS over the right STG, individuals with PD exhibited long-term improvement (2–10 weeks) in their phonetics score of Dysarthric Profile that was correlated with resting-state STG-SM1 functional connectivity. Therefore, there is mixed evidence for the benefits of TMS intervention for PD dysarthria.

Among several possible resources of the discrepancies across studies investigating the efficacy of TMS for PD dysarthria are choice of stimulation protocol and assessment of treatment outcomes. High-frequency rTMS has been the most frequent protocol used to increase cortical excitability of the DLPFC, SM1, STG, and M1 for the treatment of PD dysarthria (Dias et al., 2006; Hartelius et al., 2010; Eliasova et al., 2013; Brabenec et al., 2019). A series of neuroimaging studies on individuals with PD, however, have shown an overactivation of the DLPFC, premotor cortex (PMC), supplementary motor area (SMA), and insula during speech production (Liotti et al., 2003; Pinto et al., 2004; Arnold et al., 2014) and enhanced cortical event-related potential (ERP) P2 responses to voice pitch perturbations that were source-localized in the left STG, inferior parietal lobule (IPL), inferior frontal gyrus (IFG), and PMC (Huang et al., 2016). It is therefore plausible that PD dysarthria may result from hyperactivity in the cortical speech motor networks, and thus inhibiting, rather than enhancing, cortical excitability of those regions may reduce this hyperactivity and restore normal levels of brain activation to produce beneficial effects. This hypothesis is in line with the findings of improved articulatory functions in individuals with PD following low-frequency but not high-frequency rTMS over the right STG (Brabenec et al., 2019). On the other hand, acoustic (e.g., fundamental frequency or *f*_o_, intensity, and formant frequency) and/or perceptual (e.g., phonetics score and speech intelligibility) analyses of PD speech were generally used to evaluate the outcome of TMS intervention (Dias et al., 2006; Eliasova et al., 2013; Brabenec et al., 2019, 2021). These speech characteristics, however, reflect a broad range of mental processes, including voluntary attempts to compensate for the symptoms of PD. As mentioned, abnormalities in the integration of auditory feedback with vocal motor control in PD, characterized by overcompensation for voice pitch and loudness perturbations (Liu et al., 2012; Chen et al., 2013; Huang et al., 2016; Mollaei et al., 2016), have been regarded as significant contributors to PD dysarthria (Sapir, 2014). Thus, an examination of the neurobehavioral correlates that support auditory-motor control of vocal production may uncover the effects of TMS intervention on PD dysarthria from the perspective of sensorimotor integration.

In the present study, we examined whether and, if so, how abnormalities in auditory-vocal integration in individuals with PD can be modulated by neuronavigated continuous theta burst stimulation (c-TBS) over the left SMA. As a specific form of rTMS protocol, c-TBS inhibits the cortical excitability for up to 60 min after less than 1-min stimulation (Huang et al., 2005). The left SMA was chosen as the stimulation target in the present study because it is reciprocally connected with the laryngeal motor cortex for vocal motor command execution (Simonyan and Horwitz, 2011) and plays a central role in the initiation, monitoring, and timing control of speech production (Tourville and Guenther, 2011; Hertrich et al., 2016). Also, the left SMA has been identified as an important motor component of the speech monitoring network (Riecker et al., 2005) and to be active when healthy individuals compensate for perturbations in voice auditory feedback (Zarate and Zatorre, 2008; Behroozmand et al., 2015). Lesions in this region result in speech motor disorders, such as impaired involuntary or spontaneous vocalization and acquired dysfluencies (Jonas, 1981; Ziegler et al., 1997). Moreover, individuals with PD have shown hyperactivity in the SMA during speech production (Liotti et al., 2003; Pinto et al., 2004; Arnold et al., 2014), and a reduction of SMA activation was accompanied by improved speech scores of the UPDRS as a result of subthalamic nucleus (STN) stimulation (Pinto et al., 2004). Thus, c-TBS over the left SMA of individuals with PD may lead to a functional normalization of the speech motor systems that produce beneficial effects on their auditory-motor control of vocal production.

The intervention outcome was assessed using the frequency-altered feedback (FAF) paradigm (Burnett et al., 1998), which involves participants hearing their voice *f*_o_ unexpectedly shifted upward or downward during vocal production. We evaluated the vocal and ERP responses (P1-N1-P2) to pitch perturbations in auditory feedback after applying active or sham c-TBS over the left SMA. Individuals with PD have been found to show larger vocal compensations for pitch perturbations and/or greater ERP P2 responses relative to healthy controls (Liu et al., 2012; Chen et al., 2013; Huang et al., 2016; Mollaei et al., 2016; Li et al., 2021). This vocal overcompensation, however, returned to normal when they vocalized the sounds with external auditory cueing (Huang et al., 2019) or received voice treatment with LSVT^®^ LOUD (Li et al., 2021). Therefore, we hypothesized that individuals with PD following c-TBS over the left SMA would likewise exhibit reduced vocal and cortical ERP responses to pitch perturbations that reflect beneficial effects on their abnormalities in auditory-vocal integration.

## Materials and methods

### Subjects

A group of 16 Chinese-speaking adults (6 women and 10 men; mean age: 64.88 ± 9.88 years) diagnosed as idiopathic PD according to the United Kingdom Parkinson’s disease Society Brain Bank (Hughes et al., 1992) participated in the present study (see details in Table 1). They were included in the present study based on the following criteria: no contraindication for magnetic resonance imaging (MRI) and TMS, no dementia or psychiatric abnormalities, no neurological diseases other than PD, no experience with musical training, and no history of neurosurgical treatment or speech therapy. Individuals with PD participated in the experiment within 1–2 h after taking their regular antiparkinsonian medication. They did not show any wearing-off phenomena and/or L-dopa-induced dyskinesias. A group of 16 neurologically normal participants was recruited as healthy controls and pairwise-matched with individuals with PD on age and sex (6 women and 10 men; mean age: 64.25 ± 6.95 years; *t* = 0.207, d.f. = 30, *p* = 0.837). All participants passed a binaural hearing screening at thresholds of 40 dB hearing level or less for 500, 1,000, 2,000, and 4,000 Hz. The research protocol was approved by the Institutional Review Board of The First Affiliated Hospital of Sun Yat-sen University, and all participants provided their written informed consent.

**TABLE 1:** Clinical and demographic data of PD patients.

ID	Gender	Age (ys)	Disease duration (ys)	H-Y stage	UPDRS-II	UPDRS-III	MMSE
1	M	73	17	2.5	12	18	27
2	F	68	7	1.5	15	26	30
3	F	64	4	2	11	16	27
4	M	53	2	1.5	4	23	29
5	M	63	10	3	15	31	30
6	F	73	5	1.5	6	11	30
7	M	63	11	3	22	26	24
8	M	42	2	1.5	5	9	30
9	F	76	3	1.5	11	12	30
10	M	48	12	2.5	17	38	30
11	M	74	8	2	16	28	30
12	F	70	4	2.5	13	30	30
13	F	65	3	1.5	10	21	30
14	M	73	3	2	14	23	30
15	M	61	6	2.5	7	48	26
16	M	72	5	1.5	1	9	29

PD, Parkinson’s disease; M, male; F, female; ys, years; H-Y Stage, Hoehn and Yahr stage, UPDRS-II, the Unified Parkinson’s Disease Rating Scale part II; UPDRS-III, the Unified Parkinson’s Disease Rating Scale part III; MMSE, Mini-Mental Status Examination.

### Magnetic resonance imaging data acquisition

Prior to the c-TBS experiment, high-resolution anatomical images were acquired from all participants using a 3T MRI scanner (Siemens, Germany) to precisely define the stimulation target. A T1-weighted magnetization-prepared rapid gradient-echo (MPRAGE) sequence was used during the scanning with the following parameters: repetition time (TR) = 2,000 ms, echo time (TE) = 1.76 ms, slice thickness = 0.8 mm, voxel size = 0.8 × 0.8 × 0.8 mm^3^, field of view (FOV) = 260 × 260 mm^2^, and 224 sagittal slices.

### Neuronavigated transcranial magnetic stimulation

Magnetic stimulation was administered using a CCY-I TMS instrument (YIRUIDE Co., Wuhan, China) equipped with a 7-cm-outer-diameter figure-of-eight coil. Prior to the c-TBS intervention, the left M1 was stimulated with single*-*pulse TMS to determine the resting motor threshold (RMT) by recording the motor evoked potentials (MEPs) of each individual from the right first dorsal interosseous (FDI) muscles. The RMT was defined as the lowest stimulus intensity sufficient to elicit an MEP ≥ 50 μV in 5 out of 10 consecutive trials in the resting right FDI (Groppa et al., 2012). A standard c-TBS protocol that consisted of 600 pulses in a theta burst pattern (bursts of 3 pulses at 50 Hz repeated every 200 ms; Huang et al., 2005) was applied over the left SMA at 80% of RMT (Grossheinrich et al., 2009). The location of the left SMA (MNI coordinates: *x* = –3, *y* = –2, and *z* = 58; Bolzoni et al., 2015) was determined by loading individual anatomical MRI data into a neuronavigation software (Visor 2.0, ANT Neuro, Netherlands) with a Polaris Spectra motion tracking system (NDI, Canada). These coordinates were slightly modified based on individual brain anatomical landmarks to ensure correct positioning over left SMA. In order to rule out any spread of current into the contralateral SMA when TMS was applied over one SMA, the coil was orientated medio-laterally with the handle pointing toward the right hemisphere to stimulate the left SMA (White et al., 2013). Active c-TBS was delivered by placing the coil closely tangential to the skull surface. Sham stimulation was delivered with the coil 90° tilted away from the target with one wing of the coil touching the scalp. The order of active or sham c-TBS over the left SMA was counterbalanced across all participants, with active and sham sessions occurring on separate days at least 48 h apart (Brabenec et al., 2019). Healthy participants served as controls to determine the degree of impairment and improvement of auditory-vocal integration in individuals with PD and thus did not receive active or sham c-TBS over the left SMA.

### Experimental design

One FAF-based vocal production experiment began immediately after applying active or sham c-TBS over the left SMA to individuals with PD. Healthy controls performed the same experiment as well. All participants were instructed to produce the vowel /u/ for about 2–3 s while hearing their voice pseudo-randomly pitch-shifted upward or downward twice by 200 cents (200 ms duration; 100 cents = 1 semitone). The first pitch perturbation occurred after a random delay of 1,500–2,500 ms relative to the vocal onset, and the second stimulus was presented after an inter-stimulus interval of 700–1,000 ms. All participants were required to take a break of 2–3 s between consecutive vocalizations to avoid vocal fatigue. Each participant produced 100 consecutive vocalizations, leading to 100 trials for + 200 cents perturbations and 100 trials for –200 cents perturbations. The vocal production experiment was conducted with the same experimental parameters after individuals with PD received active or sham c-TBS.

### Apparatus

The vocal production experiment was conducted in a sound-attenuated room. First, the voice signals were transduced through a dynamic microphone (DM2200, Takstar Inc.) and sent to an Eventide Eclipse Harmonizer through a MOTU Ultralite Mk3 Firewire audio interface. A MIDI software program (Max/MSP v.5.0 by Cycling 74) was developed to control the Eventide Eclipse Harmonizer to pitch-shift the voice signals with preset parameters. Meanwhile, transistor-transistor logic (TTL) control pulses were generated by this program to mark the onset of each pitch perturbation. Finally, the pitch-shifted voice signals were amplified by an ICON Neo Amp headphone amplifier and played back to participants through inserted earphones (ER-1, Etymotic Research Inc.). The original and pitch-shifted voice signals as well as the TTL control pulses were digitized by a PowerLab A/D converter (model ML880, AD Instruments) and recorded at 10 kHz using LabChart software (v.7.0, AD Instruments).

Simultaneously, the EEG signals were scalp-recorded using a 64-electrode Geodesic Sensor Net (Electrical Geodesics Inc.), amplified by a high-input impedance NetAmps 300 amplifier (Electrical Geodesics Inc.), and recorded at a sampling frequency of 1 kHz using NetStation software (v.4.5, Electrical Geodesics Inc.). Since the amplifier accepts scalp-electrode impedances up to 40–60 kΩ, the impedance levels of individual sensors were kept below 50 kΩ throughout the recording (Ferree et al., 2001). The EEG signals across all channels were referenced to the vertex (Cz) during the recording (Ferree et al., 2001). An experimental DIN synch cable sent the TTL control pulses to the EEG recording system for the synchronization of the voice and EEG signals.

### Data analyses

The behavioral measurement, including the peak magnitude and peak latency of vocal responses to pitch perturbations, was performed in a custom-developed IGOR PRO software program (v.6.0 by Wavemetrics Inc.) that has been previously described in detail (Huang et al., 2019). In brief, the voice *f*_o_ contours in Hertz were extracted and converted to the cent scale using the following formula: cents = 100 × [12 × log_2_(*f*_o_/reference)] [reference = 195.997 Hz (G3)]. They were then segmented into epochs ranging from –200 to +700 ms relative to the perturbation onset and visually inspected for trial-by-trial artifact rejection. Overall, 81% of the individual trials were regarded as artifact-free trials. The trials were averaged and baseline-corrected (–200 to 0 ms) to generate an overall vocal response for each condition. The magnitude and latency of a vocal response were separately measured as the maximum or minimum value in cents and the corresponding time in milliseconds when the voice *f*_o_ contour reached its peak value.

The offline analyses of EEG signals were performed using NetStation software. They were band-pass filtered between 1 and 20 Hz, segmented into epochs using a window of –200 to +500 ms relative to the perturbation onset, and submitted to an artifact detection procedure to exclude those trials with voltage values that exceeded ± 55 μv of the moving average over an 80-ms window from further analysis. A trial-by-trial visual inspection was additionally performed to ensure that bad trials were appropriately rejected. Individual electrodes that contained artifacts in more than 20% of the epochs were rejected, and files that contained more than 10 bad channels were marked bad. Finally, artifact-free individual trials were re-referenced to the average of the electrodes on each mastoid, averaged, and baseline-corrected (–200 to 0 ms) to generate an overall ERP response to pitch perturbations. Since the cortical P1, N1, and P2 responses to pitch perturbations were prominently pronounced in the frontal and central regions (Behroozmand et al., 2009; Scheerer et al., 2013), we chose 24 electrodes in three regions of interest (ROI) for statistical analysis: frontal area, including AF3, AFz, AF4, F5, F3, F1, Fz, F2, F4, and F6; fronto-central area, including FC5, FC3, FC1, FCz, FC2, FC4, and FC6; central area, including C5, C3, C1, Cz, C2, C4, and C6. The amplitudes and latencies of the P1, N1, and P2 components were extracted from the averaged ERPs for each ROI.

### Source localization

The sLORETA software^1^ (Fuchs et al., 2002) was used to localize the neural generators of the P1, N1, and P2 responses that differed between active and sham stimulation for individuals with PD. This method partitions the intracerebral volume into 6,239 cortical gray matter voxels at a 5-mm spatial resolution and calculates the standardized current density in a realistic standardized head model within the MNI152 template (Mazziotta et al., 2001). In the present study, the voxel-based sLORETA images were computed based on the averaged ERPs within 5 ms time windows centered at the maximal global field power peaks in the P1, N1, and P2 time windows and compared between active and sham stimulations using voxel-wise randomization tests with 10,000 permutations. Multiple comparisons were corrected at a whole-brain level based on the statistical non-parametric mapping. The voxels with significant differences (for corrected *p* < 0.05) were specified in MNI coordinates and Brodmann areas (BA). The results were superimposed on an anatomical template in BrainNet Viewer (Xia et al., 2013).

### Statistical analyses

The values of vocal and ERP responses were submitted to SPSS (v.20.0) for statistical analysis. For individuals with PD, the magnitudes and latencies of vocal responses were subjected to two-way RM-ANOVAs, including within-subject factors of perturbation direction (–200 vs. +200 cents) and stimulation session (active vs. sham c-TBS). The amplitudes and latencies of the P1, N1, and P2 responses were subjected to three-way RM-ANOVAs, including within-subject factors of perturbation direction, stimulation session, and electrode site (frontal, fronto-central, and central). In addition, mixed-design ANOVAs were used to compare the differences in the vocal and ERP responses between individuals with PD following active/sham stimulation and healthy controls across the conditions. Subsidiary RM-ANOVAs were performed if any higher-order interactions between these variables were significant. Bonferroni correction was used for multiple comparisons in *post hoc* analyses. Probability values for multiple degrees of freedom were corrected using the Greenhouse–Geisser correction factor if the assumption of Mauchly’s test of Sphericity was violated. In addition, effect sizes indexed by partial η^2^ were calculated to quantify the proportion of variance.

## Results

### Behavioral findings

**FIGURE 1:**
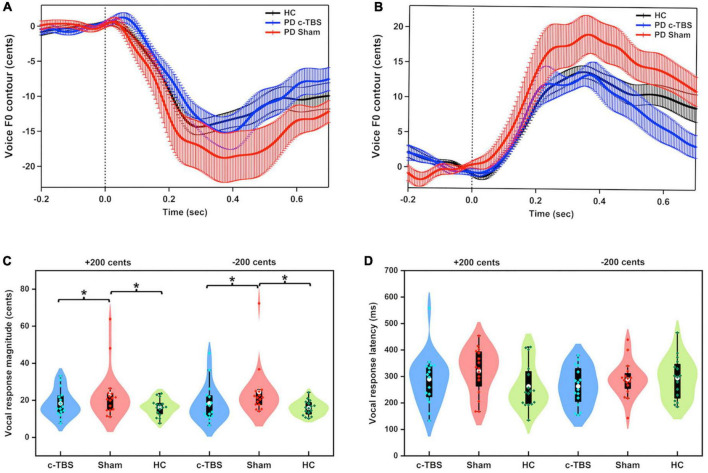
**(A,B)** Grand-averaged voice *f*_o_ contours in response to pitch perturbations of ± 200 cents in individuals with PD following active (blue solid lines) and sham (red solid lines) c-TBS over the left SMA and healthy controls (black solid lines). Vertical bars across the contours indicate the standard errors of the mean vocal responses. Vertical dashed lines indicate the onset of the pitch perturbation. **(C,D)** Violin plots of the magnitudes and latencies of vocal responses to ± 200 cents in individuals with PD following active (blue) and sham stimulation (red) and healthy controls (green). The shape of the violin shows the kernel density estimate of the data. The white dots and box plots represent the medians and range from the first to third quartiles of the data sets. The blue, red, and green dots represent the individual vocal responses to pitch perturbations. The asterisks indicate significant differences across the conditions.

Figure 1 shows the grand-averaged voice *f*_o_ responses to pitch perturbations of ± 200 cents for individuals with PD following active and sham c-TBS over the left SMA and healthy controls. A two-way RM-ANOVA conducted on the peak magnitudes of vocal responses in individuals with PD revealed a significant main effect of the stimulation session [*F*(1, 15) = 17.916, *p* = 0.001, partial η^2^ = 0.544], showing that active c-TBS over the left SMA led to smaller vocal compensations than sham stimulation (see Figure 1C). The main effect of perturbation direction [*F*(1, 15) = 0.034, *p* = 0.857] and its interaction with stimulation session [*F*(1, 15) = 0.365, *p* = 0.555], however, did not reach significance. Regarding the peak times of vocal responses, there were no significant main effects of stimulation session [*F*(1, 15) = 1.960, *p* = 0.182] and perturbation direction [*F*(1, 15) = 1.822, *p* = 0.197] (see Figure 1D). Their interaction was not significant either [*F*(1, 15) = 0.001, *p* = 0.975].

A two-way mixed-design ANOVA showed significantly larger magnitudes of vocal responses for individuals with PD receiving sham stimulation than for healthy controls [*F*(1, 30) = 7.540, *p* = 0.010, partial η^2^ = 0.201] (see Figure 1C). The main effect of perturbation direction [*F*(1, 30) = 0.292, *p* = 0.593] and its interaction with group [*F*(1, 30) = 0.338, *p* = 0.565] were not significant. However, after receiving active c-TBS over the left SMA, individuals with PD were not significantly different from the healthy controls in the magnitudes of vocal responses [*F*(1, 30) = 0.331, *p* = 0.569]. The main effect of perturbation direction [*F*(1, 30) = 0.165, *p* = 0.688] and its interaction with group [*F*(1, 30) = 0.140, *p* = 0.711] were not significant.

Regarding the peak times of vocal responses, there was no significant difference between individuals with PD receiving sham stimulation and healthy controls [*F*(1, 30) = 1.290, *p* = 0.265]. The main effect of perturbation direction [*F*(1, 30) = 0.001, *p* = 0.980] and its interaction with group [*F*(1, 30) = 3.643, *p* = 0.066] did not reach significance. Similarly, the peak times of vocal responses were not significantly different between individuals with PD receiving active c-TBS over the left SMA and healthy controls [*F*(1, 30) = 0.006, *p* = 0.940] and between upward and downward perturbations [*F*(1, 30) = 0.015, *p* = 0.904]. The interaction between these two factors was not significant [*F*(1, 30) = 2.382, *p* = 0.133].

### Event-related potential findings

**FIGURE 2:**
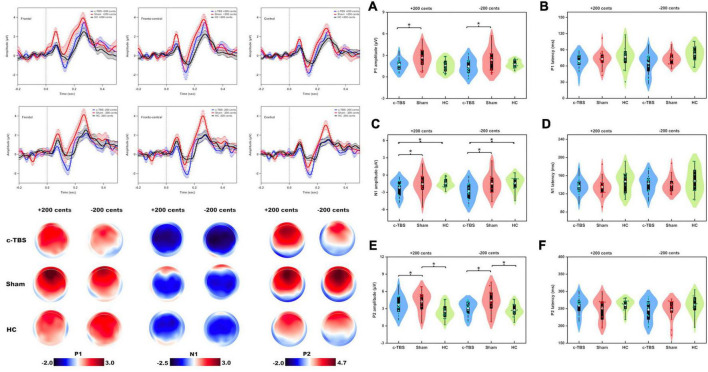
*Top left*: Grand-averaged ERPs to pitch perturbations of ± 200 cents in the frontal, fronto-central, and central regions in individuals with PD following active (blue solid lines) and sham (red solid lines) c-TBS over the left SMA and healthy controls (black solid lines). Vertical bars across the contours indicate the standard errors of the mean ERPs. Vertical dashed lines indicate the onset of the pitch perturbation. *Bottom left*: Topographical distribution maps of the P1, N1, and P2 amplitudes in responses to pitch perturbations of ± 200 cents in individuals with PD following active and sham stimulation and healthy controls. *Right Panel:* Violin plots of the amplitudes and latencies of the P1 **(A,B)**, N1 **(C,D)**, and P2 **(E,F)** responses to pitch perturbations of ± 200 cents in individuals with PD following active (blue) and sham (red) stimulation and healthy controls (green). The white dots and box plots represent the medians and range from the first to third quartiles of the data sets. The blue, red, and green dots represent the individual ERP responses to pitch perturbations. The asterisks indicate significant differences across the conditions.

Figure 2 shows the grand-averaged ERPs and topographical distributions, as well as the violin plots of the P1, N1, and P2 responses to pitch perturbations across the group and stimulation session. A three-way RM-ANOVA conducted on the P1 amplitudes in individuals with PD revealed a significant main effect of stimulation session [*F*(1, 15) = 11.679, *p* = 0.004, partial η^2^ = 0.438], where c-TBS over the left SMA led to smaller P1 responses than sham stimulation (see Figure 2A). Also, a significant main effect of electrode site [*F*(2, 30) = 9.269, *p* = 0.006, partial η^2^ = 0.382] led to smaller P1 responses at the central electrodes as compared to the frontal (*p* = 0.024) and fronto-central electrodes (*p* = 0.010). However, the main effect of perturbation direction [*F*(1, 15) = 1.738, *p* = 0.207] and interactions between any of the three factors (*p* > 0.06) were not significant. Regarding the P1 latencies, individuals with PD exhibited no significant main effects of stimulation session [*F*(1, 15) = 3.055, *p* = 0.101], perturbation direction [*F*(1, 15) = 0.175, *p* = 0.682], and electrode site [*F*(2, 30) = 0.121, *p* = 0.787] (see Figure 2B). Their interactions were also not significant (*p* > 0.2).

A three-way mixed-design ANOVA showed no significant differences in the P1 amplitudes between individuals with PD receiving sham stimulation and healthy controls [*F*(1, 30) = 3.659, *p* = 0.066]. However, there was a significant main effect of the electrode site [*F*(2, 58) = 12.448, *p* = 0.001, partial η^2^ = 0.300], showing larger P1 amplitudes at the frontal electrodes than at the fronto-central (*p* = 0.020) and central electrodes (*p* = 0.005). Also, there were larger P1 amplitudes at the fronto-central electrodes that at the central electrodes (*p* = 0.006). The main effect of perturbation direction [*F*(1, 30) = 0.219, *p* = 0.643] and the interactions between any of the three factors were not significant (*p* > 0.07). Regarding the P1 latencies, there were no significant main effects of group [*F*(1, 30) = 1.769, *p* = 0.194], perturbation direction [*F*(1, 30) = 0.576, *p* = 0.454], and electrode site [*F*(2, 58) = 0.683, *p* = 0.445]. Their interactions were not significant either (*p* > 0.5).

As well, no significant differences in the P1 amplitudes were found between individuals with PD following c-TBS over the left SMA and healthy controls [*F*(1, 30) = 0.105, *p* = 0.748] and between upward and downward perturbations [*F*(1, 30) = 0.029, *p* = 0.867]. There was a significant main effect of the electrode site [*F*(2, 58) = 20.046, *p* < 0.001, partial η^2^ = 0.401], showing smaller P1 amplitudes at the central electrodes than at the frontal (*p* < 0.001) and fronto-central electrodes (*p* < 0.001). The interactions between any of the three factors were not significant (*p* > 0.1). Regarding the P1 latencies, the main effects of group [*F*(1, 30) = 2.902, *p* = 0.099], perturbation direction [*F*(1, 30) = 0.849, *p* = 0.364], and electrode site [*F*(2, 58) = 0.173, *p* = 0.742], as well as their interactions, (*p* > 0.6) were not significant.

A three-way RM-ANOVA conducted on the N1 amplitudes in individuals with PD showed that c-TBS over the left SMA led to significantly more negative N1 responses than sham stimulation [*F*(1, 15) = 8.115, *p* = 0.012, partial η^2^ = 0.351] (see Figure 2C). A significant main effect of the electrode site [*F*(2, 30) = 16.939, *p* < 0.001, partial η^2^ = 0.530] was also found, showing less negative N1 responses at the frontal electrodes when compared to the fronto-central (*p* < 0.001) and central electrodes (*p* = 0.005). However, the main effect of perturbation direction [*F*(1, 15) = 1.443, *p* = 0.248] and interactions between any of the three factors (*p* > 0.09) were not significant. In addition, the N1 latencies did not vary significantly as a function of stimulation session [*F*(1, 15) = 0.145, *p* = 0.709], perturbation direction [*F*(1, 15) = 1.240, *p* = 0.283], and electrode site [*F*(2, 30) = 1.553, *p* = 0.233] (see Figure 2D). Their interactions were also not significant (*p* > 0.2).

A three-way mixed-design ANOVA revealed no significant differences in the N1 amplitudes between individuals with PD receiving sham stimulation and healthy controls [*F*(1, 30) = 0.082, *p* = 0.776] and between upward and downward pitch perturbations [*F*(1, 30) = 0.018, *p* = 0.893]. There was a significant main effect of the electrode site [*F*(2, 58) = 22.425, *p* < 0.001, partial η^2^ = 0.428], showing more negative N1 responses at the frontal electrodes when compared to the fronto-central (*p* < 0.001) and central electrodes (*p* = 0.001). The interactions between any of the three factors were not significant (*p* > 0.05). Regarding the N1 latencies, there were no significant main effects of group [*F*(1, 30) = 1.780, *p* = 0.192], perturbation direction [*F*(1, 30) = 0.539, *p* = 0.469], and electrode site [*F*(2, 58) = 0.439, *p* = 0.624], as well as their interactions (*p* > 0.1).

In contrast, individuals with PD receiving c-TBS over the left SMA produced significantly more negative N1 amplitudes than healthy controls [*F*(1, 30) = 7.067, *p* = 0.012, partial η^2^ = 0.191] (see Figure 2C). Also, a significant main effect of the electrode site [*F*(2, 58) = 16.335, *p* < 0.001, partial η^2^ = 0.353] led to more negative N1 responses at the frontal electrodes when compared to the fronto-central (*p* < 0.001) and central electrodes (*p* = 0.010). The main effect of perturbation direction [*F*(1, 30) = 1.657, *p* = 0.208] and interactions between any of the three factors were not significant (*p* > 0.1). Regarding the N1 latencies, there were no significant main effects of group [*F*(1, 30) = 1.162, *p* = 0.290], perturbation direction [*F*(1, 30) = 0.989, *p* = 0.328], and electrode site [*F*(2, 58) = 1.614, *p* = 0.209]. Their interactions were also not significant (*p* > 0.2).

A three-way RM-ANOVA conducted on the P2 amplitudes in individuals with PD showed that c-TBS over the left SMA led to significantly smaller P2 responses than sham stimulation [*F*(1, 15) = 13.292, *p* = 0.002, partial η^2^ = 0.470] (see Figure 2E). There was also a significant main effect of the electrode site [*F*(2, 30) = 22.031, *p* < 0.001, partial η^2^ = 0.595], which was driven by smaller P2 responses at the central electrodes as compared to the frontal (*p* = 0.001) and fronto-central electrodes (*p* < 0.001). The main effect of perturbation direction [*F*(1, 15) = 0.993, *p* = 0.335] and the interactions between any of the three factors (*p* > 0.1) were not significant. Regarding the P2 latencies, the main effects of stimulation condition [*F*(1, 15) = 0.850, *p* = 0.371], perturbation direction [*F*(1, 15) = 1.283, *p* = 0.275], and electrode site [*F*(2, 30) = 0.504, *p* = 0.514] as well as their interactions (*p* > 0.1) were not significant (see Figure 2F).

A three-way mixed-design ANOVA showed that individuals with PD receiving sham stimulation produced larger P2 responses than healthy controls [*F*(1, 30) = 9.797, *p* = 0.004, partial η^2^ = 0.246] (see Figure 2E). There was also a significant main effect of the electrode site [*F*(2, 58) = 30.140, *p* < 0.001, partial η^2^ = 0.501], showing larger P2 amplitudes at the central electrodes as compared to the frontal (*p* < 0.001) and fronto-central (*p* < 0.001) electrodes. The main effect of perturbation direction [*F*(1, 30) = 0.557, *p* = 0.461] and the interactions between any of the three factors were not significant (*p* > 0.05). Regarding the P2 latencies, there were no significant main effects of group [*F*(1, 30) = 2.619, *p* = 0.116], perturbation direction [*F*(1, 30) = 0.076, *p* = 0.784], and electrode site [*F*(2, 58) = 0.452, *p* = 0.6590]. Their interactions were not significant (*p* > 0.1).

In contrast, individuals with PD receiving c-TBS over the left SMA and healthy controls did not show significant differences in the P2 amplitudes [*F*(1, 30) = 3.183, *p* = 0.085]. There was a significant main effect of the electrode site [*F*(2, 58) = 38.215, *p* < 0.001, partial η^2^ = 0.560], showing smaller P2 amplitudes at the central electrodes than at the frontal (*p* < 0.001) and fronto-central electrodes (*p* < 0.001). Also, larger P2 amplitudes at the frontal electrodes were found when compared to the fronto-central electrodes (*p* = 0.014). The main effect of perturbation direction [*F*(1, 30) = 0.652, *p* = 0.426] and the interactions between any of the three factors were not significant (*p* > 0.1). Regarding the P2 latencies, there were no significant main effects of group [*F*(1, 30) = 0.978, *p* = 0.331], perturbation direction [*F*(1, 30) = 1.035, *p* = 0.317], and electrode site [*F*(2, 58) = 0.211, *p* = 0.728]. Their interactions were not significant (*p* > 0.2).

### Individual variability in continuous theta burst stimulation (c-TBS) effects

**FIGURE 3:**
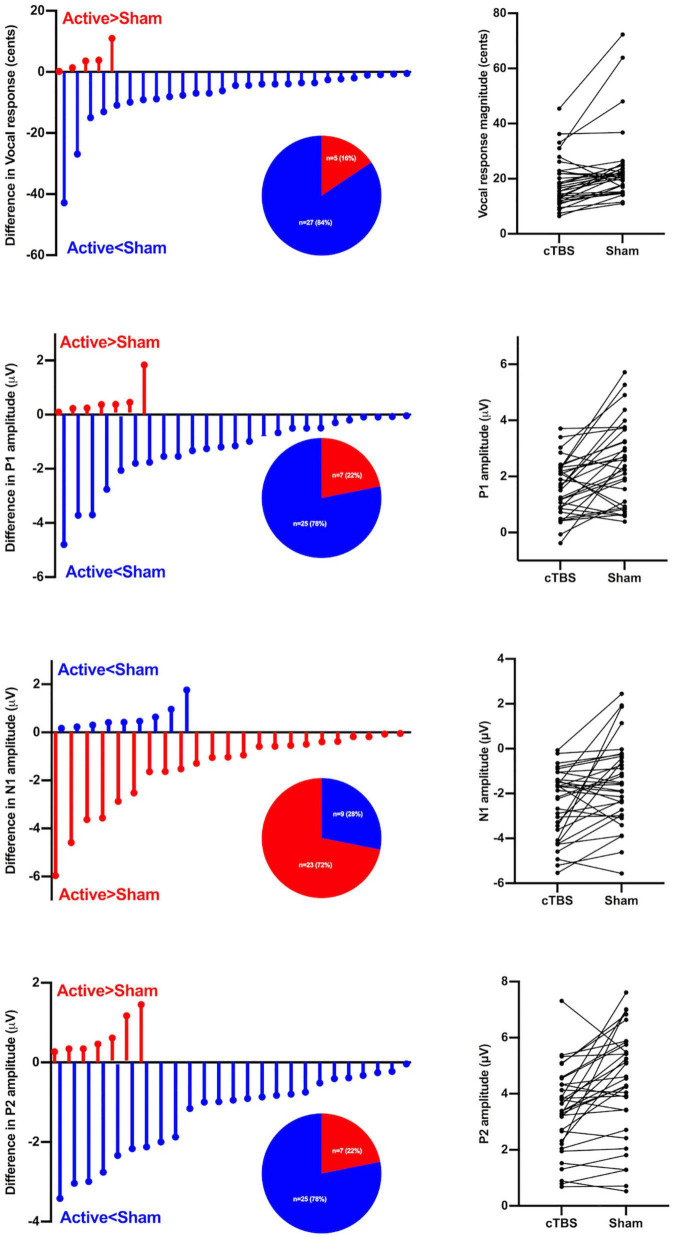
*Left panel*: Individual variability in the direction of left SMA c-TBS effects on vocal pitch regulation in individuals with PD, as reflected by differences in the vocal, P1, N1, and P2 responses to pitch perturbations of ± 200 cents between active and sham conditions. Active > Sham represents a decrease in vocal, P1, and P2 responses and an increase in N1 responses following c-TBS over the left SMA, and Sham > Active represents the opposite effects. The pie charts show the distributions of Active > Sham and Sham > Active responses across the conditions. *Right panel*: Individual vocal, P1, N1, and P2 responses to pitch perturbations of ± 200 cents between active and sham conditions in individuals with PD. Each line represents how an individual subject’s vocal or ERP (P1, N1, P2) response under c-TBS over the left SMA compares to that under sham stimulation.

Figure 3 shows the distribution of left SMA c-TBS effects on the vocal, P1, N1, and P2 responses to pitch perturbations in individuals with PD between active and sham stimulation. Active > Sham represents a decrease of vocal, P1, and P2 responses and an increase of N1 responses, while Sham > Active represents the opposite effects. Of the 32 behavioral/neural responses to upward and downward pitch perturbations produced by 16 individuals with PD, 84% of the vocal responses, 78% of the P1 responses, and 78% of the P2 responses decreased and 72% of the N1 responses increased after active c-TBS over the left SMA, reflecting differences in the direction of c-TBS effects on the neurobehavioral processing of vocal pitch errors. In addition, individual variability for this direction effect was also illustrated by the lines that connected the vocal and ERP responses in the active and sham stimulations across the participants.

### Source reconstruction findings

**FIGURE 4:**
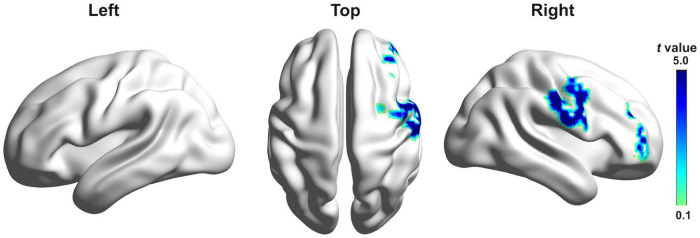
Grand-averaged sLORETA-based statistical non-parametric maps comparing the standardized current densities between active and sham c-TBS over the left SMA in the P2 time window. Results are projected onto lateral and top three-dimensional views of a standard anatomical template. Positive *t*-values indicate reduced brain activity caused by active stimulation (corrected *p* < 0.05).

Figure 4 shows estimated current density source maps that display cortical regions where individuals with PD exhibited significantly reduced P2 responses to pitch perturbations in voice auditory feedback following active vs. sham c-TBS over the left SMA. Table 2 lists the anatomical description and the MNI coordinates corresponding to these brain regions. Reduced P2 responses received contributions from a complex network, including the right inferior frontal gyrus (IFG; BA 46/10, *p* = 0.0001), middle frontal gyrus (MFG; BA 46, *p* = 0.0009), precentral gyrus (PrCG; BA 6, *p* = 0.0043), post-central gyrus (PoCG; BA 43/3, *p* = 0.0184), and insula (BA 13; *p* = 0.0269). Although systematic changes in the P1 and N1 amplitudes were found as a result of c-TBS over the left SMA, different levels of current density for these two components did not reach significance and therefore are not illustrated.

**TABLE 2:** sLORETA *t*-statistics on log-transformed data.

Condition	BA	Brain region	*t*-value	X	Y	Z	*p*
c-TBS vs. Sham	46/10	Right IFG	–5.472	45	35	15	0.0001
	46	Right MFG	–4.993	45	30	20	0.0009
	6	Right PrCG	–4.610	40	–10	35	0.0043
	43/3	Right PoCG	–4.169	65	–15	30	0.0184
	13	Right Insula	–4.057	50	–25	20	0.0269

### Brain–behavior relationship

**FIGURE 5:**
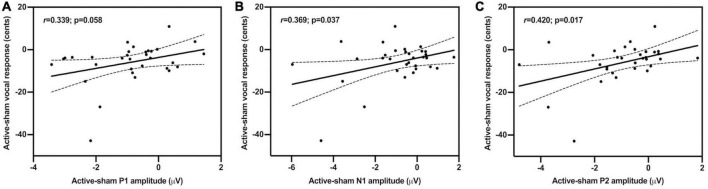
Scatter plots illustrating correlations between the active-sham differences in the magnitudes of vocal compensations and the amplitudes of the P1 **(A**); *r* = 0.339, *p* = 0.058, N1 **(B)**; *r* = 0.369, *p* = 0.037, and P2 **(C)**; *r* = 0.420, *p* = 0.017 responses to pitch perturbations.

In order to investigate the relationship between changes in vocal motor behavior and cortical brain activity induced by c-TBS over the left SMA in individuals with PD, regression analyses were performed by correlating the active-sham differences between the magnitudes of vocal responses and the amplitudes of the three ERP components. As shown in Figure 5, the active-sham magnitudes of the vocal responses were significantly correlated with the active-sham amplitudes of the N1 (*r* = 0.369, *p* = 0.037) and P2 (*r* = 0.420, *p* = 0.017) responses. That is, greater suppression of vocal responses was associated with greater enhancement of N1 responses and suppression of P2 responses, suggesting that changes in cortical brain activity induced by c-TBS over the left SMA contributed significantly to normalization of vocal pitch regulation in individuals with PD. However, this correlation was marginally significant in the active-sham differences between the magnitudes of vocal responses and the amplitudes of the P1 responses (*r* = 0.339, *p* = 0.058).

## Discussion

The present study investigated whether abnormalities in auditory-vocal integration associated with PD can be modulated by neuronavigated c-TBS over the left SMA. The results showed that individuals with PD exhibited smaller vocal compensations for pitch perturbations paralleled by smaller cortical P1 and P2 responses and larger N1 responses following c-TBS over the left SMA when compared to sham stimulation. Source reconstruction revealed contributions of the right IFG, MFG, PrCG, PoCG, and insula to reduced P2 responses induced by c-TBS over the left SMA. Notably, suppression of vocal compensations was significantly correlated with enhancement of N1 amplitudes and suppression of P2 amplitudes. In addition, as compared to healthy controls, individuals with PD exhibited a normalization of abnormally enhanced vocal and P2 responses to pitch perturbations after receiving c-TBS over the left SMA. These findings provide the first neurobehavioral evidence for the beneficial effects of c-TBS over the left SMA on impaired auditory-vocal integration in PD.

Consistent with our hypothesis, applying c-TBS over left SMA in individuals with PD decreased their abnormal vocal responses to pitch perturbations. This is in line with recent findings of decreased vocal compensations for pitch perturbations when individuals with PD vocalized with external auditory cueing (Huang et al., 2019) or received intensive voice treatment with LSVT^®^ LOUD (Li et al., 2021). As well, while replicating earlier reports of abnormally enhanced vocal compensations for pitch perturbations in individuals with PD (Liu et al., 2012; Chen et al., 2013; Huang et al., 2016; Mollaei et al., 2016), the present study found a normalization of this abnormality as a result of c-TBS over their left SMA. Similarly, individuals with PD following LSVT^®^ LOUD produced normalized and reduced vocal compensations that were significantly correlated with their improved vocal loudness during passage reading (Li et al., 2021). These findings suggest that the observed decrease and normalization of vocal compensation in individuals with PD may reflect an improvement of auditory-vocal integration induced by c-TBS over the left SMA that allows them to correctly perceive and appropriately correct mismatches between their intended and actual vocal output.

Moreover, these behavioral effects were paralleled by systematic changes in cortical brain activity in individuals with PD, as reflected by reduced P1 and P2 responses and enhanced N1 responses to pitch perturbations following c-TBS over the left SMA. Similarly, other PD studies showed that multiple sessions of low-frequency rTMS over the right STG led to enhanced resting-state functional connectivity between the right STG and parahippocampal gyrus (Brabenec et al., 2019) and increased activation of the SM1 and caudate nucleus (Brabenec et al., 2021). These two studies also reported significant correlations between improved speech articulation and enhanced right STG activation during sentence reading (Brabenec et al., 2019) and resting-state STG-SM1 functional connectivity (Brabenec et al., 2021), which is in line with our results showing that reduced vocal compensations were predicted by enhanced N1 responses and reduced P2 responses. These findings provide evidence that low-frequency rTMS or c-TBS over sensory or motor regions can lead to improvement in speech motor skills in PD.

In addition, the present study showed high inter-individual variability in the vocal and ERP responses to pitch perturbations for individuals with PD following active and sham stimulation. Moreover, not all behavioral and neural responses were modulated in the same direction: following c-TBS over the left SMA, 78–84% of vocal, P1, and P2 responses were reduced and 72% of N1 responses were enhanced, while the others showed the opposite pattern (see Figure 3). Such inter-individual variability has also been reported in previous empirical and clinical studies that investigated the TMS effects on motor and cognitive behaviors (Lopez-Alonso et al., 2014; Corp et al., 2020; Lin et al., 2022), depending on many factors such as stimulation protocol, brain structure, hormonal levels, and genetic polymorphisms (Polania et al., 2018). Addressing inter-individual variability of TMS effects has crucial implications for improving the efficacy of this non-invasive brain stimulation approach for the treatment of motor speech disorders in PD.

### Potential mechanisms of the efficacy of SMA c-TBS

The present study showed systematic changes in the neurobehavioral responses to vocal pitch errors when individuals with PD received active vs. sham c-TBS over the left SMA, highlighting the essential role of this region in vocal motor control. In the DIVA model, the SMA is hypothesized to be a central component of the feedforward control system controlling the initiation of speech motor commands (Tourville and Guenther, 2011). Dysfunctional activation of the SMA has been consistently identified in individuals with PD, particularly showing hyperactivation of this region during speech production (Liotti et al., 2003; Pinto et al., 2004; Arnold et al., 2014). As such, individuals with PD are impaired in the feedforward control of speech production, showing abnormally reduced adaptive responses to predictable voice *f*_o_ and speech *F*_1_ perturbations (Mollaei et al., 2013; Abur et al., 2018). Speech production involves a dynamic balance between feedback and feedforward control (Civier et al., 2010), and impairment in one type of control leads to a shift of the balance toward the other. Accordingly, individuals with PD may have to increase their reliance on auditory feedback as a consequence of feedforward control impairment during speech production (Chen et al., 2013; Huang et al., 2016), resulting in abnormally enhanced vocal compensations for pitch perturbations (Liu et al., 2012; Chen et al., 2013; Mollaei et al., 2016; Huang et al., 2019) paralleled by hyperactivity in the sensory and motor regions (Huang et al., 2016). In light of this point, c-TBS over the left SMA in individuals with PD may lead to a partial functional restoration of SMA that increases reliance on feedforward control and/or decreases reliance on feedback control, which in turn results in reduced and normalized vocal compensations for pitch perturbations.

Consistently, the observed changes in the cortical P1-N1-P2 responses reflect the neural impact of c-TBS over the left SMA on vocal pitch regulation in individuals with PD. It has been suggested the P1 component is responsible for the earlier detection of deviant auditory feedback in an all-or-nothing manner (Scheerer et al., 2013), while the P2 component reflects the later cortical processing of auditory-motor integration (Liu et al., 2020). Specifically, individuals with PD following c-TBS over the left SMA exhibited decreased P2 responses that were significantly correlated with decreased vocal compensations and source-localized in the right IFG, MFG, PrCG, PoCG, and insula, which is in line with other studies showing the multiple neural generators of the P2 responses in the fronto-temporo-parietal regions (Huang et al., 2016; Guo et al., 2017; Liu et al., 2020). This finding is consistent with the DIVA model that proposes a right-lateralized feedback control of speech production (Golfinopoulos et al., 2011; Tourville and Guenther, 2011). A number of empirical findings have also shown significant contributions of these right-sided regions to auditory feedback control of vocal/speech production (Toyomura et al., 2007; Tourville et al., 2008; Behroozmand et al., 2015; Kort et al., 2016; Guo et al., 2017). Moreover, the high-gamma activity of the right IFG has been shown to be significantly correlated with vocal compensations for pitch perturbations (Kort et al., 2016). Clinically, this right-sided activity pattern was also observed in individuals with PD following voice treatment with LSVT^®^ LOUD, showing increased activity in the right DLPFC, basal ganglia, and insula (Liotti et al., 2003), and a significant correlation between improved vocal loudness and increased activity in the right IFG and MFG (Narayana et al., 2010). More importantly, no significant differences were found in the P1 and P2 amplitudes between individuals with PD following active SMA c-TBS and healthy controls. Therefore, reduced and normalized P1 and P2 responses elicited by c-TBS over the left SMA in individuals with PD may reflect a functional reorganization of speech motor networks that resulted in decreased reliance on auditory feedback or increased reliance on feedforward control to facilitate the online monitoring of vocal production.

In contrast, the N1 response was enhanced following c-TBS over the left SMA in individuals with PD as compared to sham stimulation and healthy controls. This component reflects higher-level cortical encoding of auditory pitch or phonemic quality in the primary and secondary auditory cortices (Chait et al., 2004) and is sensitive to the size, direction, and timing of vocal pitch perturbations (Behroozmand et al., 2009; Liu et al., 2011). The enhancement of N1 may be indicative of downstream effects of c-TBS over the left SMA. For example, a recent study of 10 sessions of low-frequency rTMS over the right STG in individuals with PD showed increased activation of the SM1 and caudate nucleus during sentence reading (Brabenec et al., 2021). There is evidence for dysfunctional self-monitoring of auditory feedback in PD (Arnold et al., 2014; Railo et al., 2020). For example, N1 responses to self-produced speech were found to be less suppressed in individuals with PD compared to healthy individuals, and responses to passively heard phonemes were observed to be smaller (Railo et al., 2020). This abnormal neural response may exaggerate the mismatch between the intended and actual vocal output (i.e., larger prediction errors) and lead to larger vocal responses. Accordingly, enhancement of the N1 responses following c-TBS over the left SMA may represent a compensatory mechanism that allows individuals with PD to predict the sensory consequence of self-produced speech more precisely (i.e., smaller prediction errors), generating more feedforward commands to correct for pitch perturbations with smaller compensation magnitudes.

Several inherent limitations of the present study should be addressed. First of all, the present study reported the immediate effects of SMA c-TBS on auditory-motor integration for vocal pitch regulation in a single-session manner, limiting the assessment of treatment outcome with the measures of speech characteristics, such as vocal acoustics (e.g., *f*_o_, intensity), articulatory function, and speech intelligibility, and clinical assessment, such as voice handicap index (VHI) and visual analog scale (VAS). Also, the sample size is relatively small, although it is consistent with other studies that investigated PD-related speech disorders (Huang et al., 2016; Mollaei et al., 2016; Brabenec et al., 2019, 2021). In order to fully address the clinical efficacy and long-term effects of c-TBS for the treatment of PD dysarthria, future randomized, double-blind, sham-controlled studies are warranted in a multiple-session manner, and a comprehensive assessment of speech motor skills with larger sample sizes should be conducted in future. Second, responses to the FAF-based vocal production task were not measured for individuals with PD prior to active or sham SMA c-TBS. Asking our individuals with PD to perform the vocal production task twice (before and after c-TBS) would require them to generate a total of 200 consecutive vocalizations. Our pilot tests showed that individuals with PD experienced serious vocal fatigue and other uncomfortable issues that significantly worsened the quality of the vocal and EEG data when producing so many vocalizations. Thus, there may exist baseline variability in the vocal and EEG data between two different days (active and sham SMA c-TBS) that cannot be ruled out in the present study. In addition, passively listening to self-produced voice was not included, making it difficult to determine whether the c-TBS effects observed in individuals with PD are the result of their dysfunctions in auditory processing alone or their dysfunctions in auditory-motor integration. Further work is needed to address this question by examining the modulation of SIS by applying TMS over certain cortical regions.

In summary, the present study showed that c-TBS over the left SMA in individuals with PD led to reduced vocal compensations for pitch perturbations paralleled by reduced P1 and P2 responses and enhanced N1 responses. These findings provide the first neurobehavioral evidence for causal modulations of auditory-motor integration for vocal pitch regulation in individuals with PD by c-TBS over the left SMA, suggesting that neuronavigated c-TBS over speech motor regions may be a promising strategy to facilitate auditory-motor control of vocal production in PD.

## Data availability statement

The raw data supporting the conclusions of this article will be made available by the authors, without undue reservation.

## Ethics statement

The studies involving human participants were reviewed and approved by Institutional Review Board of The First Affiliated Hospital of Sun Yat-sen University. The patients/participants provided their written informed consent to participate in this study.

## Author contributions

GD, EW, PL, XC, and HL contributed to the design of the study. GD, MW, YL, ZG, TL, YC, and LC contributed to the acquisition and analysis of data. GD, JJ, TL, PL, XC, and HL contributed to drafting the manuscript and preparing the figures. All authors have reviewed and approved the contents of the manuscript.

## Footnotes

1
http://www.uzh.ch/keyinst/loreta.htm

## References

[B1] Abur D. Lester-Smith R. A. Daliri A. Lupiani A. A. Guenther F. H. Stepp C. E. (2018). Sensorimotor adaptation of voice fundamental frequency in Parkinson’s disease. *PLoS One* 13:e0191839. 10.1371/journal.pone.0191839 29373589 PMC5786318

[B2] Arnold C. Gehrig J. Gispert S. Seifried C. Kell C. A. (2014). Pathomechanisms and compensatory efforts related to Parkinsonian speech. *Neuroimage. Clin.* 4 82–97. 10.1016/j.nicl.2013.10.016 24319656 PMC3853351

[B3] Behroozmand R. Karvelis L. Liu H. Larson C. R. (2009). Vocalization-induced enhancement of the auditory cortex responsiveness during voice F0 feedback perturbation. *Clin. Neurophysiol.* 120 1303–1312. 10.1016/j.clinph.2009.04.022 19520602 PMC2710429

[B4] Behroozmand R. Shebek R. Hansen D. R. Oya H. Robin D. A. Howard M. A. III (2015). Sensory-motor networks involved in speech production and motor control: An fMRI study. *Neuroimage* 109 418–428. 10.1016/j.neuroimage.2015.01.040 25623499 PMC4339397

[B5] Bolzoni F. Bruttini C. Esposti R. Castellani C. Cavallari P. (2015). Transcranial direct current stimulation of SMA modulates anticipatory postural adjustments without affecting the primary movement. *Behav. Brain. Res.* 291 407–413. 10.1016/j.bbr.2015.05.044 26055201

[B6] Brabenec L. Klobusiakova P. Barton M. Mekyska J. Galaz Z. Zvoncak V. (2019). Non-invasive stimulation of the auditory feedback area for improved articulation in Parkinson’s disease. *Parkinsonism Relat. Disord.* 61 187–192. 10.1016/j.parkreldis.2018.10.011 30337204

[B7] Brabenec L. Klobusiakova P. Simko P. Kostalova M. Mekyska J. Rektorova I. (2021). Non-invasive brain stimulation for speech in Parkinson’s disease: A randomized controlled trial. *Brain Stimul.* 14 571–578. 10.1016/j.brs.2021.03.010 33781956

[B8] Burnett T. A. Freedland M. B. Larson C. R. Hain T. C. (1998). Voice F0 responses to manipulations in pitch feedback. *J. Acoust. Soc. Am.* 103 3153–3161. 9637026 10.1121/1.423073

[B9] Chait M. Simon J. Z. Poeppel D. (2004). Auditory M50 and M100 responses to broadband noise: Functional implications. *Neuroreport* 15 2455–2458. 10.1097/00001756-200411150-00004 15538173

[B10] Chen R. Seitz R. J. (2001). Changing cortical excitability with low-frequency magnetic stimulation. *Neurology* 57 379–380. 11502898 10.1212/wnl.57.3.379

[B11] Chen X. Zhu X. Wang E. Q. Chen L. Li W. Chen Z. (2013). Sensorimotor control of vocal pitch production in Parkinson’s disease. *Brain Res.* 1527 99–107. 23820424 10.1016/j.brainres.2013.06.030

[B12] Chou Y. H. Hickey P. T. Sundman M. Song A. W. Chen N. K. (2015). Effects of repetitive transcranial magnetic stimulation on motor symptoms in Parkinson disease: A systematic review and meta-analysis. *JAMA Neurol.* 72 432–440. 25686212 10.1001/jamaneurol.2014.4380 PMC4425190

[B13] Civier O. Tasko S. M. Guenther F. H. (2010). Overreliance on auditory feedback may lead to sound/syllable repetitions: Simulations of stuttering and fluency-inducing conditions with a neural model of speech production. *J. Fluency Disord.* 35 246–279. 10.1016/j.jfludis.2010.05.002 20831971 PMC2939043

[B14] Corp D. T. Bereznicki H. G. K. Clark G. M. Youssef G. J. Fried P. J. Jannati A. (2020). Large-scale analysis of interindividual variability in theta-burst stimulation data: Results from the ‘Big TMS Data Collaboration’. *Brain Stimul.* 13 1476–1488. 10.1016/j.brs.2020.07.018 32758665 PMC7494610

[B15] Dias A. E. Barbosa E. R. Coracini K. Maia F. Marcolm M. A. Fregni F. (2006). Effects of repetitive transcranial magnetic stimulation on voice and speech in Parkinson’s disease. *Acta Neurol. Scand.* 113 92–99. 16411969 10.1111/j.1600-0404.2005.00558.x

[B16] Duffy J. R. (2005). *Motor speech disorders: Substrates, differential diagnosis, and management.* St. Louis, MO: Mosby.

[B17] Eliasova I. Mekyska J. Kostalova M. Marecek R. Smekal Z. Rektorova I. (2013). Acoustic evaluation of short-term effects of repetitive transcranial magnetic stimulation on motor aspects of speech in Parkinson’s disease. *J. Neural Transm.* 120 597–605. 10.1007/s00702-012-0953-1 23254927

[B18] Ferree T. C. Luu P. Russell G. S. Tucker D. M. (2001). Scalp electrode impedance, infection risk, and EEG data quality. *Clin. Neurophysiol.* 112 536–544. 10.1016/s1388-2457(00)00533-2 11222977

[B19] Fitzgerald P. B. Fountain S. Daskalakis Z. J. (2006). A comprehensive review of the effects of rTMS on motor cortical excitability and inhibition. *Clin. Neurophysiol.* 117 2584–2596. 16890483 10.1016/j.clinph.2006.06.712

[B20] Fuchs M. Kastner J. Wagner M. Hawes S. Ebersole J. S. (2002). A standardized boundary element method volume conductor model. *Clin. Neurophysiol.* 113 702–712. 11976050 10.1016/s1388-2457(02)00030-5

[B21] Golfinopoulos E. Tourville J. A. Bohland J. W. Ghosh S. S. Nieto-Castanon A. Guenther F. H. (2011). fMRI investigation of unexpected somatosensory feedback perturbation during speech. *Neuroimage* 55 1324–1338. 10.1016/j.neuroimage.2010.12.065 21195191 PMC3065208

[B22] Groppa S. Oliviero A. Eisen A. Quartarone A. Cohen L. G. Mall V. (2012). A practical guide to diagnostic transcranial magnetic stimulation: Report of an IFCN committee. *Clin. Neurophysiol.* 123 858–882. 10.1016/j.clinph.2012.01.010 22349304 PMC4890546

[B23] Grossheinrich N. Rau A. Pogarell O. Hennig-Fast K. Reinl M. Karch S. (2009). Theta burst stimulation of the prefrontal cortex: Safety and impact on cognition, mood, and resting electroencephalogram. *Biol. Psychiatry* 65 778–784. 10.1016/j.biopsych.2008.10.029 19070834

[B24] Guo Z. Wu X. Li W. Jones J. A. Yan N. Sheft S. (2017). Top-down modulation of auditory-motor integration during speech production: The role of working memory. *J. Neurosci.* 37 10323–10333. 28951450 10.1523/JNEUROSCI.1329-17.2017 PMC6596622

[B25] Hallett M. (2007). Transcranial magnetic stimulation: A primer. *Neuron* 55 187–199. 17640522 10.1016/j.neuron.2007.06.026

[B26] Hartelius L. Svantesson P. Hedlund A. Holmberg B. Revesz D. Thorlin T. (2010). Short-term effects of repetitive transcranial magnetic stimulation on speech and voice in individuals with Parkinson’s disease. *Folia Phoniatr. Logop.* 62 104–109. 20424465 10.1159/000287208

[B27] Hertrich I. Dietrich S. Ackermann H. (2016). The role of the supplementary motor area for speech and language processing. *Neurosci. Biobehav. Rev.* 68 602–610. 27343998 10.1016/j.neubiorev.2016.06.030

[B28] Huang X. Chen X. Yan N. Jones J. A. Wang E. Q. Chen L. (2016). The impact of Parkinson’s disease on the cortical mechanisms that support auditory-motor integration for voice control. *Hum. Brain Mapp.* 37 4248–4261. 10.1002/hbm.23306 27400999 PMC6867337

[B29] Huang X. Fan H. Li J. Jones J. A. Wang E. Q. Chen L. (2019). External cueing facilitates auditory-motor integration for speech control in individuals with Parkinson’s disease. *Neurobiol. Aging* 76 96–105. 10.1016/j.neurobiolaging.2018.12.020 30710834

[B30] Huang Y. Z. Edwards M. J. Rounis E. Bhatia K. P. Rothwell J. C. (2005). Theta burst stimulation of the human motor cortex. *Neuron* 45 201–206. 15664172 10.1016/j.neuron.2004.12.033

[B31] Hughes A. J. Daniel S. E. Kilford L. Lees A. J. (1992). Accuracy of clinical diagnosis of idiopathic Parkinson’s disease: A clinico-pathological study of 100 cases. *J. Neurol. Neurosurg. Psychiatry* 55 181–184. 10.1136/jnnp.55.3.181 1564476 PMC1014720

[B32] Jonas S. (1981). The supplementary motor region and speech emission. *J. Commun. Disord.* 14 349–373. 7287912 10.1016/0021-9924(81)90019-8

[B33] Kort N. S. Cuesta P. Houde J. F. Nagarajan S. S. (2016). Bihemispheric network dynamics coordinating vocal feedback control. *Hum. Brain Mapp.* 37 1474–1485. 10.1002/hbm.23114 26917046 PMC6867418

[B34] Li Y. Tan M. Fan H. Wang E. Q. Chen L. Li J. (2021). Neurobehavioral effects of LSVT ^®^ LOUD on auditory-vocal integration in Parkinson’s disease: A preliminary study. *Front. Neurosci.* 15:624801. 10.3389/fnins.2021.624801 33716652 PMC7952622

[B35] Lin Q. Chang Y. Liu P. Jones J. A. Chen X. Peng D. (2022). Cerebellar continuous theta burst stimulation facilitates auditory-vocal integration in spinocerebellar ataxia. *Cereb. Cortex* 32 455–466. 10.1093/cercor/bhab222 34240142

[B36] Liotti M. Ramig L. O. Vogel D. New P. Cook C. I. Ingham R. J. (2003). Hypophonia in Parkinson’s disease: Neural correlates of voice treatment revealed by PET. *Neurology* 60 432–440. 10.1212/wnl.60.3.432 12578924

[B37] Liu D. Dai G. Liu C. Guo Z. Xu Z. Jones J. A. (2020). Top-down inhibitory mechanisms underlying auditory-motor integration for voice control: Evidence by TMS. *Cereb. Cortex* 30 4515–4527. 10.1093/cercor/bhaa054 32147719

[B38] Liu H. Meshman M. Behroozmand R. Larson C. R. (2011). Differential effects of perturbation direction and magnitude on the neural processing of voice pitch feedback. *Clin. Neurophysiol.* 122 951–957. 20869305 10.1016/j.clinph.2010.08.010 PMC3151676

[B39] Liu H. Wang E. Q. Verhagen Metman L. Larson C. R. (2012). Vocal responses to perturbations in voice auditory feedback in individuals with Parkinson’s disease. *PLoS One* 7:e33629. 10.1371/journal.pone.0033629 22448258 PMC3308986

[B40] Lopez-Alonso V. Cheeran B. Rio-Rodriguez D. Fernandez-Del-Olmo M. (2014). Inter-individual variability in response to non-invasive brain stimulation paradigms. *Brain Stimul.* 7 372–380. 24630849 10.1016/j.brs.2014.02.004

[B41] Mazziotta J. Toga A. Evans A. Fox P. Lancaster J. Zilles K. (2001). A probabilistic atlas and reference system for the human brain: International Consortium for Brain Mapping (ICBM). *Philos. Trans. R. Soc. Lond. B Biol. Sci.* 356 1293–1322. 10.1098/rstb.2001.0915 11545704 PMC1088516

[B42] Mollaei F. Shiller D. M. Baum S. R. Gracco V. L. (2016). Sensorimotor control of vocal pitch and formant frequencies in Parkinson’s disease. *Brain Res.* 1646 269–277. 10.1016/j.brainres.2016.06.013 27288701 PMC4975987

[B43] Mollaei F. Shiller D. M. Gracco V. L. (2013). Sensorimotor adaptation of speech in Parkinson’s disease. *Mov. Disord.* 28 1668–1674. 23861349 10.1002/mds.25588 PMC3812368

[B44] Moreau C. Pinto S. (2019). Misconceptions about speech impairment in Parkinson’s disease. *Mov. Disord.* 34 1471–1475. 10.1002/mds.27791 31307114

[B45] Narayana S. Fox P. T. Zhang W. Franklin C. Robin D. A. Vogel D. (2010). Neural correlates of efficacy of voice therapy in Parkinson’s disease identified by performance-correlation analysis. *Hum. Brain Mapp.* 31 222–236. 10.1002/hbm.20859 19639554 PMC2811230

[B46] Pascual-Leone A. Valls-Sole J. Wassermann E. M. Hallett M. (1994). Responses to rapid-rate transcranial magnetic stimulation of the human motor cortex. *Brain* 117(Pt 4) 847–858. 7922470 10.1093/brain/117.4.847

[B47] Pinto S. Thobois S. Costes N. Le Bars D. Benabid A. L. Broussolle E. (2004). Subthalamic nucleus stimulation and dysarthria in Parkinson’s disease: A PET study. *Brain* 127 602–615. 10.1093/brain/awh074 14736753

[B48] Polania R. Nitsche M. A. Ruff C. C. (2018). Studying and modifying brain function with non-invasive brain stimulation. *Nat. Neurosci.* 21 174–187. 29311747 10.1038/s41593-017-0054-4

[B49] Railo H. Nokelainen N. Savolainen S. Kaasinen V. (2020). Deficits in monitoring self-produced speech in Parkinson’s disease. *Clin. Neurophysiol.* 131 2140–2147. 10.1016/j.clinph.2020.05.038 32682241

[B50] Ramig L. O. Sapir S. Fox C. M. Countryman S. (2001). Changes in vocal intensity following intensive voice treatment (LSVT) in individuals with Parkinson diseases: A comparison with untreated patients and normal age-matched controls. *Mov. Disord.* 16 79–83. 10.1002/1531-8257(200101)16:1<79::aid-mds1013>3.0.co;2-h 11215597

[B51] Riecker A. Mathiak K. Wildgruber D. Erb M. Hertrich I. Grodd W. (2005). fMRI reveals two distinct cerebral networks subserving speech motor control. *Neurology* 64 700–706. 10.1212/01.WNL.0000152156.90779.89 15728295

[B52] Rusz J. Cmejla R. Tykalova T. Ruzickova H. Klempir J. Majerova V. (2013). Imprecise vowel articulation as a potential early marker of Parkinson’s disease: Effect of speaking task. *J. Acoust. Soc. Am.* 134 2171–2181. 10.1121/1.4816541 23967947

[B53] Sapir S. (2014). Multiple factors are involved in the dysarthria associated with Parkinson’s disease: A review with implications for clinical practice and research. *J. Speech Lang. Hear. Res.* 57 1330–1343. 10.1044/2014_JSLHR-S-13-0039 24686571

[B54] Sapir S. Ramig L. Fox C. (2008). Speech and swallowing disorders in Parkinson disease. *Curr. Opin. Otolaryngol. Head Neck Surg.* 16 205–210. 18475072 10.1097/MOO.0b013e3282febd3a

[B55] Sapir S. Spielman J. L. Ramig L. O. Story B. H. Fox C. (2007). Effects of intensive voice treatment (the Lee Silverman Voice Treatment [LSVT]) on vowel articulation in dysarthric individuals with idiopathic Parkinson disease: Acoustic and perceptual findings. *J. Speech Lang. Hear. Res.* 50 899–912. 17675595 10.1044/1092-4388(2007/064)

[B56] Scheerer N. E. Behich J. Liu H. Jones J. A. (2013). ERP correlates of the magnitude of pitch errors detected in the human voice. *Neuroscience* 240 176–185. 23466810 10.1016/j.neuroscience.2013.02.054

[B57] Simonyan K. Horwitz B. (2011). Laryngeal motor cortex and control of speech in humans. *Neuroscientist* 17 197–208. 21362688 10.1177/1073858410386727 PMC3077440

[B58] Skodda S. Rinsche H. Schlegel U. (2009). Progression of dysprosody in Parkinson’s disease over time–a longitudinal study. *Mov. Disord.* 24 716–722. 10.1002/mds.22430 19117364

[B59] Tourville J. A. Guenther F. H. (2011). The DIVA model: A neural theory of speech acquisition and production. *Lang. Cogn. Process.* 26 952–981. 10.1080/01690960903498424 23667281 PMC3650855

[B60] Tourville J. A. Reilly K. J. Guenther F. H. (2008). Neural mechanisms underlying auditory feedback control of speech. *Neuroimage* 39 1429–1443. 18035557 10.1016/j.neuroimage.2007.09.054 PMC3658624

[B61] Toyomura A. Koyama S. Miyamaoto T. Terao A. Omori T. Murohashi H. (2007). Neural correlates of auditory feedback control in human. *Neuroscience* 146 499–503. 17395381 10.1016/j.neuroscience.2007.02.023

[B62] White O. Davare M. Andres M. Olivier E. (2013). The role of left supplementary motor area in grip force scaling. *PLoS One* 8:e83812. 10.1371/journal.pone.0083812 24391832 PMC3877107

[B63] Xia M. Wang J. He Y. (2013). BrainNet Viewer: A network visualization tool for human brain connectomics. *PLoS One* 8:e68910. 10.1371/journal.pone.0068910 23861951 PMC3701683

[B64] Zarate J. M. Zatorre R. J. (2008). Experience-dependent neural substrates involved in vocal pitch regulation during singing. *Neuroimage* 40 1871–1887. 10.1016/j.neuroimage.2008.01.026 18343163

[B65] Ziegler W. Kilian B. Deger K. (1997). The role of the left mesial frontal cortex in fluent speech: Evidence from a case of left supplementary motor area hemorrhage. *Neuropsychologia* 35 1197–1208. 10.1016/s0028-3932(97)00040-7 9364490

